# Mucus carbohydrate composition correlates with scleractinian coral phylogeny

**DOI:** 10.1038/s41598-024-64828-5

**Published:** 2024-06-18

**Authors:** Bianca M. Thobor, Arjen Tilstra, Benjamin Mueller, Andreas Haas, Jan-Hendrik Hehemann, Christian Wild

**Affiliations:** 1 Department of Marine Ecology, University of Bremen, Bremen, Germany; 2 Department of Freshwater and Marine Ecology, University of Amsterdam, Amsterdam, The Netherlands; 3 CARMABI Foundation, Willemstad, Curaçao; 4 Department of Microbiology and Biogeochemistry, NIOZ Royal Netherlands Institute for Sea Research, Texel, The Netherlands; 5 Department of Marine Glycobiology, Max Planck Institute for Marine Microbiology, Bremen, Germany; 6 MARUM Centre for Marine Environmental Sciences, University of Bremen, Bremen, Germany

**Keywords:** Molecular ecology, Ecology, Carbohydrates

## Abstract

The mucus surface layer serves vital functions for scleractinian corals and consists mainly of carbohydrates. Its carbohydrate composition has been suggested to be influenced by environmental conditions (e.g., temperature, nutrients) and microbial pressures (e.g., microbial degradation, microbial coral symbionts), yet to what extend the coral mucus composition is determined by phylogeny remains to be tested. To investigate the variation of mucus carbohydrate compositions among coral species, we analyzed the composition of mucosal carbohydrate building blocks (i.e., monosaccharides) for five species of scleractinian corals, supplemented with previously reported data, to discern overall patterns using cluster analysis. Monosaccharide composition from a total of 23 species (belonging to 14 genera and 11 families) revealed significant differences between two phylogenetic clades that diverged early in the evolutionary history of scleractinian corals (i.e., complex and robust; *p* = 0.001, R^2^ = 0.20), mainly driven by the absence of arabinose in the robust clade. Despite considerable differences in environmental conditions and sample analysis protocols applied, coral phylogeny significantly correlated with monosaccharide composition (Mantel test: *p* < 0.001, R^2^ = 0.70). These results suggest that coral mucus carbohydrates display phylogenetic dependence and support their essential role in the functioning of corals.

## Introduction

The metazoan surface mucus layer (SML) is an outermost protective barrier of exposed tissues^1^, and first evolved in Cnidarians and Ctenophores^2^. Mucus consists largely of water (95%) and mucin glycoproteins (~ 3%), which have a high (50–90%) carbohydrate content in the form of glycans (oligo- and polysaccharides) attached to a protein backbone^3^, giving mucus its viscoelastic properties^2^. Traditionally, mucus has been considered to serve important functions in metazoan defense, feeding and locomotion^4^. Moreover, its role in controlling associated microbial communities is increasingly recognized^2^. Constant renewal of the SML serves a physical antimicrobial function^2,5^, and chemical defenses include adhesion (entrapment) or the prevention of adhesion (dispersal) of microbes to mucin glycans^2,6^. Changes in mucin glycan structures can reduce antimicrobial functions of mucus^7,8^, and may lead to disease^9^, highlighting the importance of mucosal carbohydrates for metazoan health.

Corals are considered model systems for metazoan evolution^2,10,11^, and scleractinian corals in particular are ecologically important due to their role as ecosystem engineers of tropical as well as cold water coral reefs^12,13^. Mucus serves especially diverse functions in corals compared to other invertebrates^14^, including protection against environmental stressors (e.g., desiccation, UV radiation, and sediment smothering), supplementation of calcification, and quenching of potentially harmful oxygen radicals (reviewed by Brown and Bythell^14^), as well as colonial integration through mucus-coordinated surface flows^15^. Despite these important functions of coral mucus, little is known about the composition of coral mucus glycans, nor their phylogenetic variation.

The structural analysis of glycans is challenging due to the lack of distinct spectroscopic signatures^16^, and mass spectra of mucin-type glycans are especially complex and notoriously difficult to interpret^17^. To our best knowledge, only two studies investigated the structure of coral mucus glycans in detail through mass spectrometry of oligosaccharides cleaved from the protein backbone of mucins^18,19^. A less challenging and more commonly used method for total carbohydrate analysis is the measurement of monosaccharide building blocks after acid hydrolysis of glycans though chromatographic methods^20^, which provides insight into the composition of carbohydrates at the cost of losing structural information^16^. Scleractinian corals from different geographic locations displayed common mucus monosaccharides^21^, and Wild et al.^22^ found conserved monosaccharide compositions of coral mucus glycans on the genus level in *Acropora* and *Fungia*.

Phylogenetic dependence (i.e., related species resemblance) is often low for carbohydrates^23,24^, due to the constant selection pressure from co-evolving pathogens^25^ and microbial degradation^26^ (i.e., Red Queen effect/arms race). In addition, several factors can contribute to inter- and intraspecific variation in coral mucus. Firstly, coral mucus release rates and/or compositions can be influenced by environmental variables like water temperature^27–29^ and nutrient enrichment^30^. Secondly, microbial communities associated with coral mucus can vary with environmental conditions^31^, potentially resulting in composition adaptations. Finally, endosymbiotic dinoflagellates of the family Symbiodiniaceae^32^ are majorly involved in mucus production^33–35^, and likely contribute to shaping mucus composition^36,37^.

This raises the question whether the previously suggested phylogenetic dependence in coral mucus carbohydrate compositions is limited to the genus level (e.g., as previously shown for *Acropora* and *Fungia*)^22^, or if it also applies to broader taxonomic groups. Scleractinian corals diverged into two main clades (i.e., “complex”, and “robust”) about 418 million years ago^38^, which have few morphological differences, but differ in some biosynthetic pathways^39,40^. We hypothesized that (1) mucus carbohydrate compositions are most different between the complex and robust clade and that (2) the phylogeny of scleractinian corals correlates with the composition of coral mucus carbohydrates, indicative for phylogenetic dependence. For the investigation, we analyzed the monosaccharide composition of hydrolized mucus glycans from five species of scleractinian corals (i.e., *Acropora cervicornis, Diploria labyrinthiformis, Meandrina meandrites*: collected in situ in the Caribbean; *Montipora digitata,* and *Montipora confusa*: grown ex situ in Bremen, Germany) and combined our results with reported literature data (total of 23 species from 14 genera and 11 families).

## Results

### Monosaccharide composition of mucosal carbohydrates

The monosaccharide compositions of mucosal carbohydrates of the five species analyzed in the present study (i.e., *A. cervicornis, D. labyrinthiformis, M. meandrites*: collected in situ on Curaçao and maintained in ambient seawater; *M. confusa*, *M. digitata*: grown in aquarium facilities in Bremen, Germany) revealed a common presence of glucosamine (GlcN) in all analyzed samples, and an absence of rhamnose (Rha), while all other monosaccharides (galactosamine, GalN; xylose, Xyl; galactose, Gal; fucose, Fuc; glucose, Glc; mannose, Man; arabinose, Ara) were only present in certain species (Table 1, Supplementary Table S1). The two Indo-Pacific sister species *M. confusa* and *M. digitata* had almost identical mucus carbohydrate compositions of GlcN (54.0 ± 1.6 and 56.9 ± 2.8 mol%) and Ara (46.0 ± 1.6 and 43.1 ± 2.8 mol%), while mucus carbohydrate compositions of *D. labyrinthiformis* and *M. meandrites* differed from *A. cervicornis*, due to high relative contribution of Fuc (47.8 ± 1.1 and 33.6 ± 7.1 vs. 2.0 ± 0.4 mol%) to total carbohydrates (see mean concentrations in Table 1). The total carbohydrate concentration of mucus from *A. cervicornis* was 9 to 16 times higher compared to the other species (ANOVA: F_(4, 9)_ = 59.2, η^2^ = 0.96, *p* < 0.001; Tukey HSD: *p* < 0.001), which enabled the detection of monosaccharides with low relative abundance.

**Table 1:** Mean concentration (mg L^−1^) ± SD of monosaccharides and total carbohydrates in hydrolyzed coral mucus of five coral species.

Species	Region and in situ vs. ex situ growth	N	GalN	Xyl	Gal	Fuc	Glc	Man	Ara	GlcN	Total
*Acropora cervicornis*	Collected in situ in the Caribbean (Curaҫao)	3	1.53 ± 0.06	0.14 ± 0.25	2.68 ± 0.10	1.04 ± 0.08	n.d	10.07 ± 3.65	22.80 ± 1.75	13.73 ± 2.82	51.98 ± 8.15
*Diploria labyrinthiformis*	Collected in situ in the Caribbean (Curaҫao)	3	0.02 ± 0.03	0.17 ± 0.30	n.d	2.60 ± 2.68	0.03 ± 0.04	1.05 ± 1.36	n.d	1.76 ± 1.69	5.62 ± 5.73
*Meandrina meandrites*	Collected in situ in the Caribbean (Curaҫao)	2	n.d	n.d	n.d	1.14 ± 0.59	0.18 ± 0.25	0.38 ± 0.03	n.d	1.79 ± 0.23	3.48 ± 1.10
*Montipora confusa*	From Indo-Pacific, grown ex situ in Bremen, Germany	3	n.d	n.d	n.d	n.d	n.d	n.d	1.39 ± 0.04	1.95 ± 0.13	3.34 ± 0.14
*Montipora digitata*	From Indo-Pacific, grown ex situ in Bremen, Germany	3	n.d	n.d	n.d	n.d	n.d	n.d	2.02 ± 0.55	3.15 ± 0.54	5.17 ± 1.18

Rhamnose was not detected (n.d.) in any sample. N = number of replicates, GalN = galactosamine, Xyl = xylose, Gal = galactose, Fuc = fucose, Glc = glucose, Man = mannose, Ara = arabinose, GlcN = glucosamine, Total = sum of all measured monosaccharides. Raw data is available in Supplementary Table S1.

### Cluster analysis including literature data

**Figure 1:**
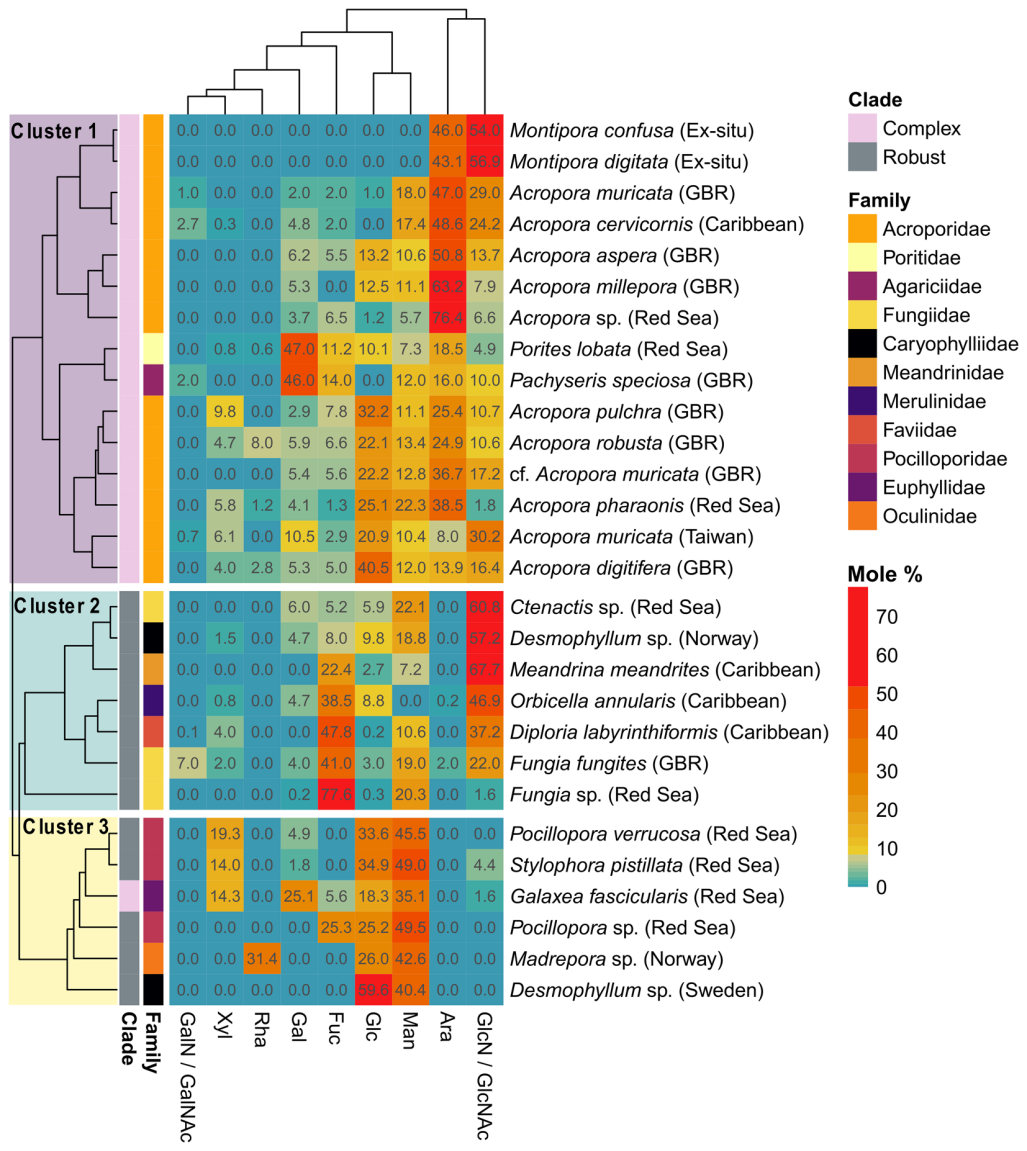
Coral mucus carbohydrate compositions form three significantly different clusters. Hierarchical cluster analysis and heatmap of relative monosaccharide compositions per coral species (mole % values are given in cells), measured in the present study and six previous studies (see Supplementary Table S2 for more detail). Dendrogram is based on Euclidean distance. GalN/GalNAc = galactosamine/*N*-acetyl-galactosamine, Xyl = xylose, Rha = rhamnose, Gal = galactose, Fuc = fucose, Glc = glucose, Man = mannose, Ara = arabinose, GlcN/GlcNAc = glucosamine /*N*-acetyl-glucosamine.

**Figure 2:**
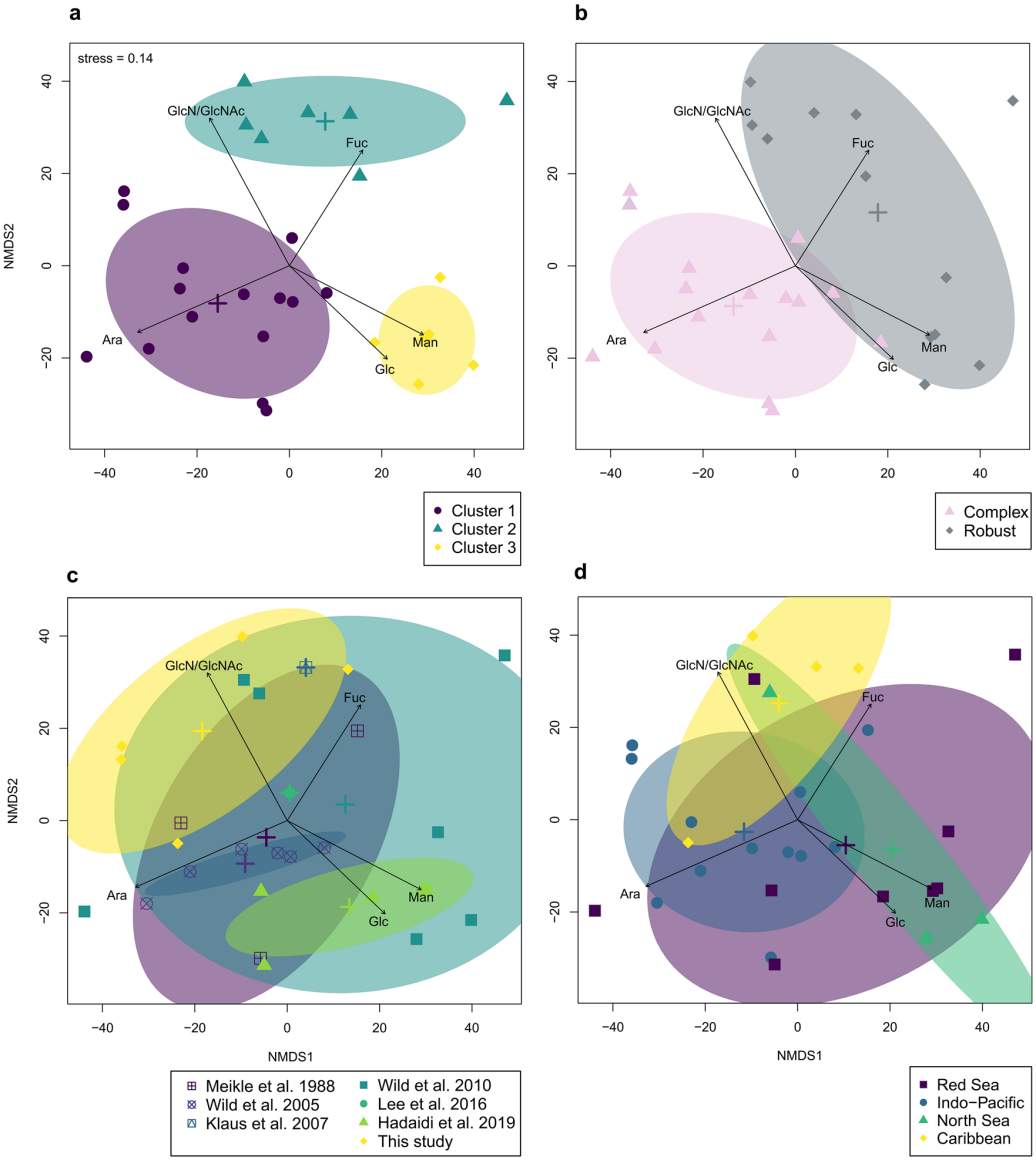
Non-metric multidimensional scaling of coral mucus carbohydrate compositions divided by (**a**) clusters established in hierarchal cluster analysis (see Fig. 1), (**b)** phylogenetic clade, (**c**) study where the data originated, and (**d**) geographic origin of specimen. Vectors for monosaccharides were only shown when significant (*p* < 0.05). All factors were significant in permutational multivariate analysis of variance, but only **a** and **b** revealed significant differences between groups in pairwise comparisons (*pairwiseAdonis*,* p* < 0.05). Plus-signs mark centroids of respective groups, and ellipses mark areas of 68% confidence. Fuc = fucose, Glc = glucose, Man = mannose, Ara = arabinose, GlcN/GlcNAc = glucosamine /N-acetyl-glucosamine.

Hierarchical cluster analysis including data from the present study (Table 1) and six previous studies (Supplementary Table S2) revealed three significantly different clusters (PERMANOVA: *F*_(2)_ = 12.9, *p* = 0.001; all pairwise comparisons: *p* = 0.003, Bonferroni adjusted; Figs. 1 and 2a) which explained 51% of variance. Overall, the monosaccharides GlcN / N-acetyl glucosamine (GlcNAc), Ara, Man, and Glc were the most common, and GalN / N-acetyl galactosamine (GalNAc) and Rha were the least common components of coral mucus carbohydrates (Fig. 1). The difference between corals of the complex and robust clade alone was also significant and explained 20% of the observed variance (PERMANOVA: *F*_(1)_ = 7.9, *p* = 0.001; Figs. 1 and 2b). Monosaccharide compositions did not differ between studies (PERMANOVA: *F*_(4)_ = 3.2, *p* = 0.033; all pairwise comparisons: *p* > 0.05; Fig. 2c), nor between geographic regions where the coral specimen originated from (PERMANOVA: *F*_(3)_ = 2.5, *p* = 0.007; all pairwise comparisons: *p* > 0.05; Fig. 2d).

**Figure 3:**
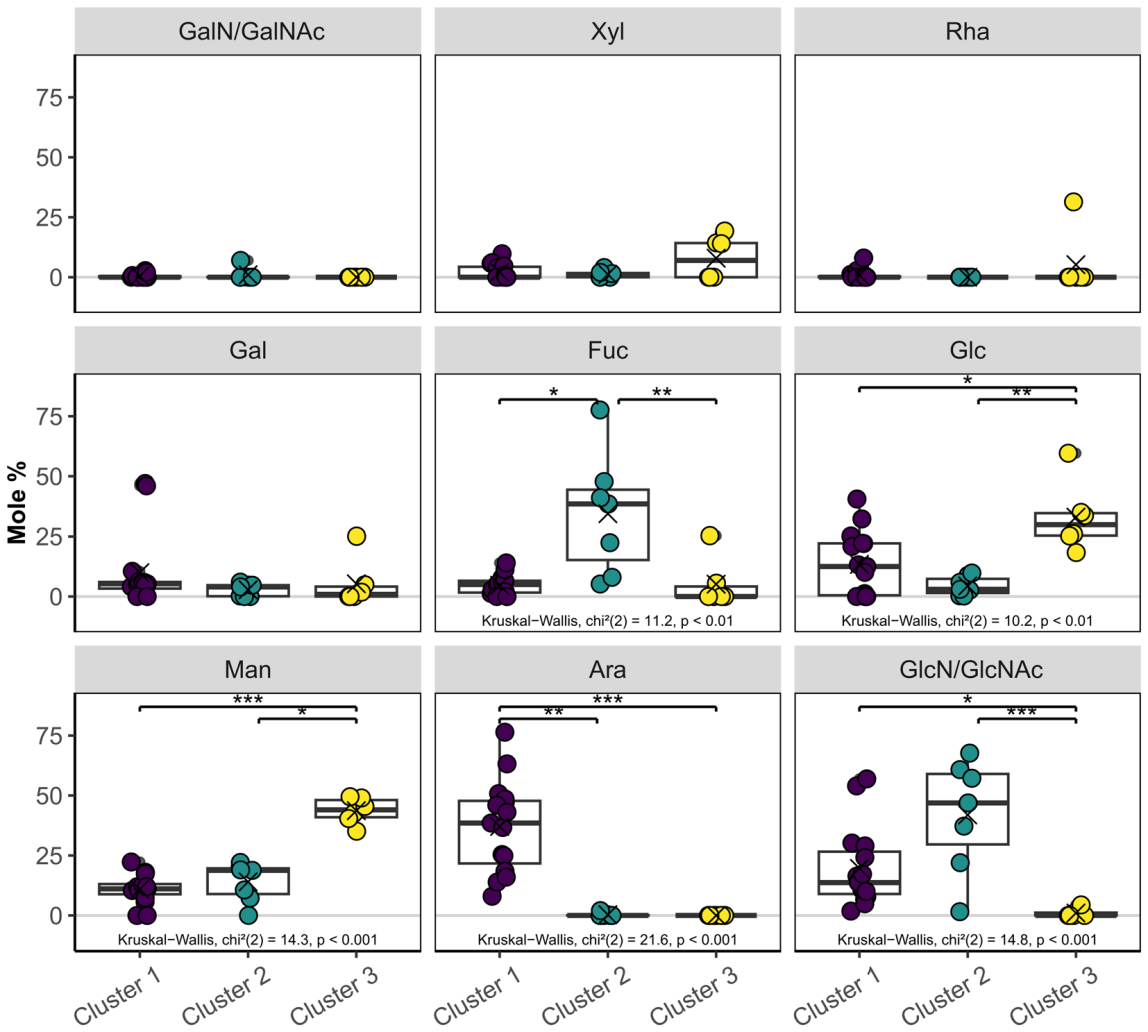
Characterization of the three clusters from hierarchical cluster analysis (Fig. 1). Comparison of relative monosaccharide contents among clusters. Asterisks indicate significant differences between clusters (*p < 0.05, **p < 0.01, ***p < 0.001, Dunn’s test, Bonferroni adjusted), where Kruskal Wallis tests were significant (results reported on the bottom of the respective panel). Boxes represent the interquartile range, with the horizontal black line indicating the median, and black crosses indicating the mean. GalN/GalNAc = galactosamine / N-acetyl-galactosamine, Xyl = xylose, Rha = rhamnose, Gal = galactose, Fuc = fucose, Glc = glucose, Man = mannose, Ara = arabinose, GlcN/GlcNAc = glucosamine /N-acetyl-glucosamine.

The first cluster exclusively included coral species of the complex clade, covering three families and four genera, and all reported measurements of the family Acroporidae (Fig. 1). The cluster was characterized by significantly more Ara compared to the two other clusters (*p* < 0.01, Dunn’s test, Bonferroni adjusted; see Fig. 3 for all Kruskal–Wallis test results), and significantly more GlcN compared to the third cluster (*p* < 0.05).

The second cluster was only composed of corals from the robust clade, covering five families and six genera, including all three measurements of the family Fungiidae (Fig. 1). Mucus carbohydrates contained significantly more Fuc than the two other clusters (*p* < 0.05), and significantly more GlcN than the third cluster (*p* < 0.001; Fig. 3).

The third cluster included all species of the family Pocilloporidae, as well as two other families of the robust clade, and *Galaxea fascicularis* of the complex clade (Fig. 1). Mucus carbohydrates contained significantly more Man (*p* < 0.05) and Glc (*p* < 0.05) than the other clusters (Fig. 3).

### Correlation between coral mucus carbohydrate composition and coral phylogeny

**Figure 4:**
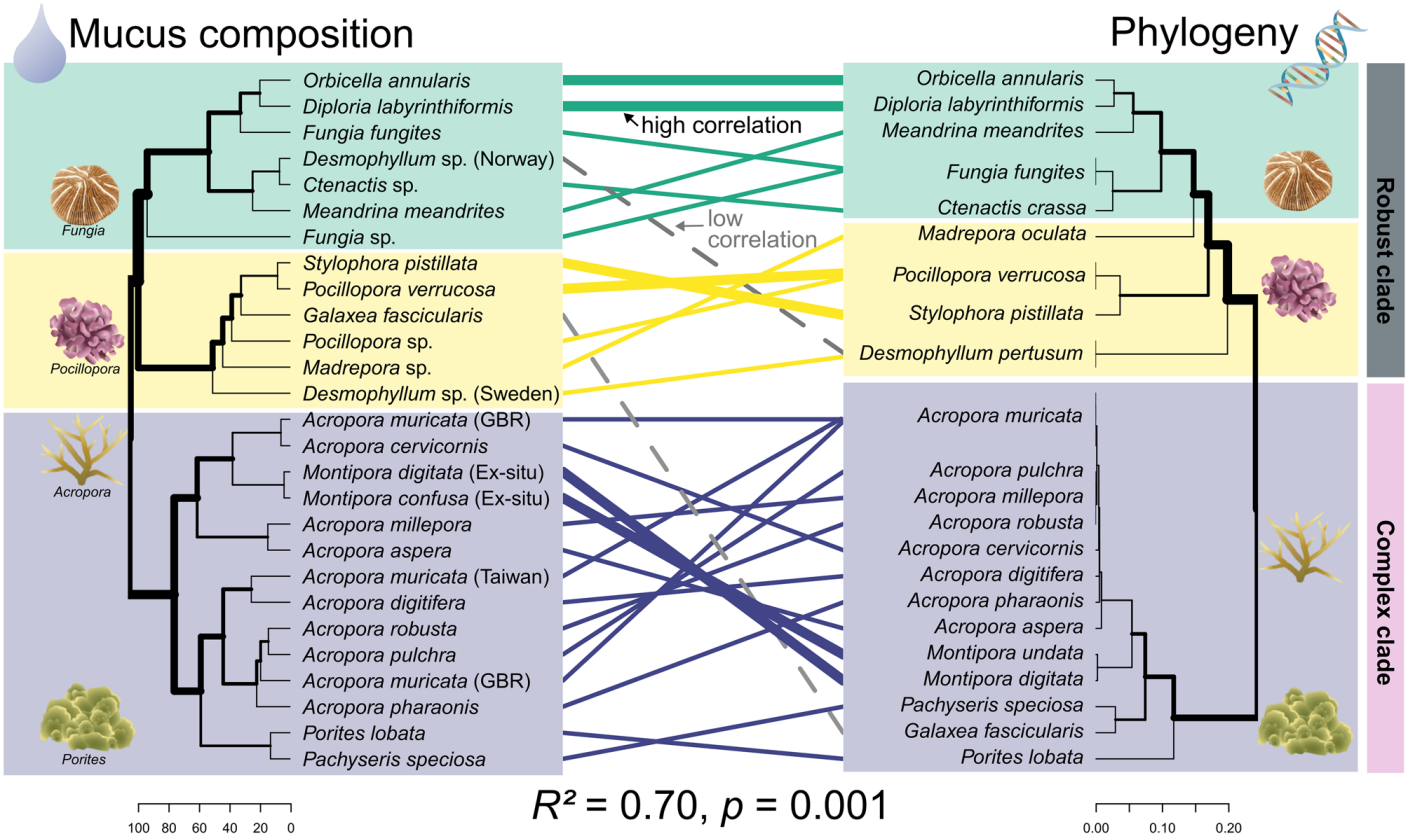
Mucus carbohydrate composition (left dendrogram, derived from Fig. 1) and phylogenetic tree (right dendrogram, based on cytochrome *c* oxidase subunit I (COI)) correlate significantly. Result of the Mantel test is given on the bottom, where the simulated *p* value is based on 999 permutations. Colors of connecting lines highlight the three main clusters of the left dendrogram. Bold lines indicate closely related species with high similarity in mucus compositions (i.e., high correlation). Grey dashed lines indicate species where the mucus carbohydrate composition reflects a different cluster compared to closely related species (i.e., low correlation). Symbols represent different scleractinian coral genera and are added for illustrational purposes. Symbol attribution: Integration and Application Network (ian.umces.edu/media-library), CC BY-SA 4.0 license.

To quantify the correlation of scleractinian coral phylogeny on coral mucus carbohydrate composition, we created a phylogenetic tree including the same species (or close sister species) used in the dendrogram for carbohydrate compositions (i.e., Fig. 1). Both dendrograms were connected with lines for visual comparison (Fig. 4), and a Mantel test was used to compare the two distance matrices, revealing a significant correlation (R = 0.70, *p* = 0.001, 999 permutations). The three clusters from the carbohydrate dendrogram were mostly reflected by the phylogenetic tree, with the exception of *Desmophyllum sp.* (Norway) and *G. fascicularis* (see grey dashed lines in Fig. 4).

For closely related species, relationships between phylogeny and mucus carbohydrate composition were more variable than on the broader taxonomic scale. *Orbicella annularis* and *D. labyrinthiformis*, *Stylophora pistillata* and *Pocillopora verrucosa,* as well as *M. digitata* and *M. confusa* were highly correlated (see bold connecting lines in Fig. 4). However, differences in mucus carbohydrate composition within the genera *Desmophyllum*, *Fungia,* and *Pocillopora* were greater than differences between closely related families (i.e., Merulinidae and Faviidae). In addition, the three available carbohydrate measurements of mucus from *Acropora muricata* (from three different studies) displayed as much variation as was observed on the family level within the Acroporidae.

## Discussion

Hierarchical cluster analysis of the combined data on coral mucus carbohydrate compositions of 23 scleractinian coral species and 11 families revealed three significantly different clusters (Figs. 1 and 2a), due to differences in the monosaccharides Ara, Fuc, GlcN/GlcNAc, Glc, and Man (Fig. 3). The absence of Ara in the robust clade mainly contributed to a significant difference between complex and robust corals, a pattern which could not be explained by differences between geographic regions where the specimen originated, nor between studies which first reported the data (Fig. 2). Finally, the dendrogram containing the three clusters correlated significantly with the phylogenetic tree of scleractinian corals (Fig. 4).

### Coral mucus carbohydrate composition may display phylogenetic dependence

Results of the present study revealed a significant correlation between coral mucus carbohydrate composition and coral phylogeny, which is more apparent at a broad taxonomic scale (i.e., between the complex and robust clades) and may indicate phylogenetic dependence of the mucosal carbohydrate building blocks (i.e., monosaccharides). Phylogenetic dependence (or phylogenetic signal) is the trend of traits being more similar between more closely related species, and can be explained by genetic drift^25^. Random mutations are suggested to lead to increasing differences in genes with increasing phylogenetic distance (i.e., the timespan since divergence of lineages)^23^.

Meikle et al.^19^, one of the few studies that analyzed the detailed glycan structure of coral mucus, revealed that mucins of *A. muricata* (then *A. formosa*^41^) are highly glycosylated through O-glycosidic links, and glycan side chains rich in Ara, Man, and GlcNAc. These same monosaccharides (i.e., Ara, Man, and GlcN/GlcNAc) were common in mucus of all *Acropora* species analyzed in the present study (Fig. 1) and were thus likely parts of mucins. The protein backbone of mucins (encoded by MUC genes) dictates the position of O-glycans, which can only be attached to the hydroxyl groups of serine or threonine^42^. The MUC genes differ between branches of the tree of life^43^, and evolutionary variation of the regions where glycosylation occurs can lead to structural and functional changes of mucins^44^. Thus, the correlation of coral mucus carbohydrate composition and coral phylogeny may indicate phylogenetic dependence in MUC genes and/or genes for enzymes involved in glycosylation. This connection could be further investigated by studying the involved genes (i.e., MUC and GT) in scleractinian corals.

Glycan structures and compositions usually do not reflect phylogenetic lineages, as glycans evolve rapidly to escape pathogens^23^ and general microbial degradation^26^ (i.e., Red Queen effect/arms race). For example, Tao et al.^24^ proposed that rather than phylogeny, selective environmental and microbial pressures shape oligosaccharide compositions (carbohydrates composed of several monosaccharides) in primate milk. Variation of host glycans is limited by the necessity to retain vital functions^45^ such as successful interactions with microbial symbionts^23^. Thus, phylogenetic dependence of carbohydrate compositions may contribute to conserving vital functions for scleractinian corals. However, monosaccharide compositions of hydrolyzed glycans lack information on the overall glycan structure^16^, and future studies should aim to capture their full molecular variation (e.g., as proposed by Bligh et al.^26^).

### Phylogenetic dependence despite various environmental and microbial pressures

The three mucus carbohydrate compositions of *A. muricata* used in this analysis originated from Taiwan^27^ and the Great Barrier Reef (GBR) in Australia^46,47^ and revealed large differences. These differences could be explained by different environmental conditions, as water temperature was shown to influence coral mucus carbohydrate composition in *A. muricata*^27^. On the other hand, Klaus et al. (2007) found no intra-specific variation across depth- and coastal pollution gradients in mucus carbohydrates of *M. annularis*^48^. Thus, more research is needed to elucidate the species-specific effects of environmental factors on coral mucus carbohydrate compositions. Furthermore, changes in microbial communities due to environmental conditions^31^ and ex situ culturing^49,50^ (i.e., for *M. digitata* and *M. confusa* colonies in the present study) may influence coral mucus compositions, and thus should be included in future studies due to expected interaction of mucosal carbohydrates with associated microbes^9,51^. Lastly, endosymbiotic Symbiodiniaceae likely influence coral mucus compositions^36,37^ and the taxonomic composition of Symbiodiniaceae communities can vary widely among and within species of scleractinian corals^52,53^. We did not analyze the Symbiodiniaceae communities associated with the investigated corals in the present study, so we do not know which taxa of endosymbionts were present. But, Symbiodiniaceae-coral associations for 64 species of 18 genera of hard corals (including the genera *Acropora*, *Galaxea*, *Pocillopora*, *Pachyseris, Porites* and *Montipora* included in the present study) differed between species, while they did not depend on phylogenetic clades (i.e., complex or robust)^54^. Arabinose is not common in animal cells^46,55^, and may be delivered to the coral host by the endosymbiotic dinoflagellates^22,46^. Arabinose characterized the mucus of corals from the complex clade, the only exception to this trend being *G. fascicularis*. The origin of Ara in the mucus of corals from the complex clade should be further investigated and studying the mucus Ara content in response to coral bleaching, or differences in dominant Symbiodiniaceae genera could reveal a link to Symbiodiniaceae metabolism. Subsequently, it could be expected that theses environmental and microbial pressures largely determine the variation in mucus carbohydrate composition and thereby overrule potential effects of phylogeny. Surprisingly, in the present study the opposite appears to be the case with coral phylogeny explaining 70% of the observed variation on the level of monosaccharide building blocks in coral mucus glycans.

### Potential caveats and limitations

Apart from the aforementioned potential effects of variations in environmental conditions, geographic locations, and ex situ vs in situ collections, the use of different sampling protocols and analytical methods have likely affected the mucus carbohydrate compositions reported in the different studies which were included in the cluster analysis (Supplementary Table S3). Coral mucus sampling was conducted either by drawing mucus from the corals’ surface with low stress^21,48^, or by removing the coral from water and catching the dripping mucus (i.e., “milking”^22,27,47^; and present study), which induces stress and may impact mucus compositions^14^. Next to mucins, mucus collected with this method may contain seawater released from the coelenteron and tissue debris^56^. Nevertheless, “milking” is an efficient way to collect relatively pure mucus samples (i.e., without environmental contamination) from corals, and is also used frequently in microbial ecology^57–59^. Additionally, carbohydrate analysis was conducted either by gas chromatography coupled with mass spectrometry (GC–MS)^21,22,46,47^, high-performance liquid chromatography with MS detection (HPLC–MS)^27^, or high-performance anion-exchange chromatography with pulsed amperometric detection (HPAEC-PAD^48^; and present study). GC–MS requires chemical alteration of sugar molecules, while HPAEC-PAD does not require this step and has a lower detection range, making it more suitable for environmental samples^60^. As coral mucus carbohydrate concentrations are generally high, the difference in detection limit between methods may be negligible, and differences in accuracy between methods are less relevant when comparing relative compositions (i.e., mol%). Furthermore, dialysis membranes used for desalination of mucus samples ranged in pore size between 0.1 and 50 kDa, and no dialysis was carried out in the present study where samples were diluted instead. The majority of carbohydrates in coral mucus are in the form of mucin glycoproteins and large heteropolysaccharides which have molecular sizes of 175–30,000 kDa^18,56,61^. Thus, these molecules should have remained in the samples with any of the used pore sizes. Finally, we acknowledge the limited number of replicates for some of the analyzed coral species, which was one for some of the early measurements^46^, and two to three for our analyses (more detail in Supplementary Table S2). Despite the potential effects of these limitations, we would like to point out that there was no significant effect of study on the monosaccharide composition (Fig. 2c). In combination with the fact that 70% of the observed variation in monosaccharide composition could be explained by coral phylogeny, this may indicate the dominating effect of phylogeny on the composition of monosaccharide building blocks in coral mucus carbohydrates.

## Conclusion

The carbohydrate compositions of coral mucus from 23 species originated from seven different studies (including the present study), and were thus likely influenced by (i) differences in environmental conditions (including in situ vs ex situ growth), (ii) associated microbiota, and (iii) sample preparation- and measurement methods. Despite these factors which can induce variation, the mucus compositions from corals of the complex and robust clade were significantly different, and coral phylogeny explained 70% of the variation. Therefore, our results indicate that coral mucus carbohydrate composition exhibits phylogenetic dependence (on the level of their monosaccharide building blocks), suggesting important functions of mucosal carbohydrates for scleractinian corals.

## Methods

### Mucus collection

Fragments of the critically endangered coral species *A. cervicornis* (n = 3) were temporarily provided by the coral restoration project Reef Renewal Curaçao to avoid any detrimental pressure on natural populations. Coral fragments were suspended on coral trees (i.e., floating pipe structures at 10 m depth) with strings and could therefore be removed and transported to the CARMABI research station without tissue damage. Colonies of *D. labyrinthiformis* (n = 3) and *M. meandrites* (n = 2) were collected from the reef in Piscadera Bay (12.121012, − 68.970380) while avoiding injury to living tissue. All corals were kept and allowed to recover for one week at a suspended artificial structure at 10 m depth in front of the CARMABI research station. Colonies were brought to a seawater flow-through aquarium in the morning, incubated in an aquarium with 22 L of filtered seawater (0.2 µm pore size) for 6 h at ambient temperature and light conditions (29.0 °C ± 0.2 SD, 101 µmol photons m^−2^ s^−1^ ± 13 SD) as part of a different study (Thobor et al., in preparation), and then again placed in the flow-through aquarium for the night. Mucus was sampled the next morning by exposing the colonies to air, and collecting the dripping mucus for two minutes in a sterile falcon tube after discarding the first 30 s, according to Wild et al.^47^. This method is also called “milking” of corals, and may result in different biochemical compositions of mucus than what is present in the surface layer when undisturbed^14^. *Montipora digitata* (n = 3) and *M. confusa* (n = 3) colonies from the Indo-Pacific were grown in the aquarium facilities of the Marine Ecology department of the University of Bremen for six years under stable conditions (water temperature: ~ 26 °C; light: ~ 100–150 µmol photons m^−2^ s^−1^; salinity: ~ 35‰; sea salt: Zoo Mix, Tropic Marin, Switzerland). Although these two species are the only ones which were not collected in situ, we decided to include them in the analysis, as the aim of the study was to investigate phylogenetic effects on mucus composition. Mucus collection was done as described above, and colonies were placed back into the aquarium. All mucus samples were stored at − 20 °C until further processing, as was done before for the analysis of carbohydrates in coral mucus samples^22,27,62^.

### Measurement of monosaccharide compositions

Mucus samples were hydrolyzed at 100 °C for 24 h by adding 50 µL of 2 M HCl to 50 µL of mucus. Afterwards, mucus was diluted by a factor of 100 by adding 20 µL of the mucus-HCl mixture to 980 µL of ultrapure water (UW). Diluted mucus samples were vortexed, and then centrifuged (15 min at 21,100×*g*), and 100 µL of the top layer were transferred into glass vials for measurement together with six calibration standards including all monosaccharides at concentrations ranging from 10 to 1000 µg L^−1^. Monosaccharide concentrations of hydrolyzed mucus were measured with a high-performance anion exchange chromatography system (Dionex ICS-5000^+^, Thermo Fisher Scientific), equipped with a PA10 column (2 × 250 mm) and PA10 guard column (both by Thermo Fisher Scientific). Monosaccharides were separated by an isocratic flow of 18 mM NaOH for 20 min. HPAEC was coupled with pulsed amperometric detection (HPAEC-PAD) as previously described^20^.

### Data preparation for hierarchical cluster analysis

Monosaccharide concentrations measured in the present study were converted to mole %, using the mean of two (*M. meandrites*) or three (all other species) replicates. Data of mucus carbohydrate compositions of 23 different scleractinian coral species, measured with varying numbers of replicates ranging from one to 36 (see Table S2 for more detail) were retrieved from six previous studies^21,22,27,46–48^. Species or genera with several reported mucus compositions from different locations or studies (i.e., *A. muricata*, *Desmophyllum sp.*) were treated separately in analyses. Only studies which could detect the nine neutral and amino sugars Fuc, Rha, GalN/GalNAc, Ara, GlcN/GlcNAc, Gal, Glc, Man, and Xyl were included (see comparison of methods used in Table S3). GlcN was pooled with its derivate GlcNAc, and GalN was pooled with GalNAc. Species names were changed to the currently accepted names, i.e., “*Montastrea annularis*” now *Orbicella annularis*^63^, “*Acropora formosa*” now *A. muricata*^41^, “*A. nobilis*” now *A. robusta*^41^, and finally “*Lophelia* sp.” now *Desmophyllum* sp.^64^. Three studies^21,47,48^ did not report the absence of GalN/GalNAc, but values were set to zero because the methods used (Table S3) enable the detection of GalN/GalNAc. Specifically, Wild et al.^22^, reported the absence of GalNAc for data measured in Wild et al.^47^ and the GC–MS analysis in Hadaidi et al.^21^ were conducted by the same analytical facility as for Wild et al.^22,47^, thus also being able to detect GalN/GalNAc. Finally, Klaus et al.^48^ used HPAEC-PAD, the same method used in the present study which can detect GalN/GalNAc^20^. Similarly, absence of Rha was not reported in two studies^27,46^ although methods used (Table S3) can detect Rha, and values were set to zero. Two studies^21,48^ reported the relative abundance of additional monosaccharides, and mole % values were adjusted accordingly for better comparison among studies. Mole % data of *Fungia* sp.^22^ was averaged from three measurements conducted in different seasons. Finally, *Stylophora* sp.^22^ was not included in the hierarchical cluster analysis of the present study, because only one monosaccharide (Glc) was detected. This was likely due to low carbohydrate concentrations in the mucus, leading to the sole detection of one monosaccharide, which was then over-estimated as contributing to 100% of carbohydrates.

### Phylogenetic tree construction

To be able to correlate the mucus carbohydrate dendrogram with coral phylogeny, a phylogenetic tree was constructed based on cytochrome *c* oxidase subunit I (COI). The COI sequences were downloaded from GenBank (see Supplementary Table S4). Sequences were loaded into Geneious Prime software (version 2023.0.3) and aligned using the Geneious Alignment tool. The phylogenetic tree was constructed based on unweighted pair group with arithmetic mean (UPGMA) with Hasegawa, Kishino, and Yano (HKY) genetic distances using the Geneious Tree Builder tool. The resulting distance matrix was exported and used to create the tanglegram (see statistical analyses section). For comparisons of unspecified genera or in case a COI sequence of a selected species was not available, COI sequences of sister species within the same genus were downloaded and used to construct the phylogenetic tree (*Genus species* mucus dendrogram vs. *Genus species* phylogeny), i.e., *Desmophyllum* sp. vs. *Desmophyllum pertusum*, *Ctenactis* sp. vs. *Ctenactis crassa*, *Fungia* sp. vs. *Fungia fungites*, *Pocillopora* sp. vs. *Pocillopora verrucosa*, *Madrepora* sp. vs. *Madrepora oculata*, and finally *Montipora confusa* vs. *Montipora undata.* These substitutes were chosen as follows: only one COI sequence was available for the genera *Desmophyllum*, *Ctenactis*, and *Madrepora*. *Montipora confusa* was substituted with the available COI sequence which was most closely related, and unspecified genera which were already present in the phylogenetic tree (i.e., *Fungia* sp. and *Pocillopora* sp.) were merged with the already present species of the respective genus.

### Statistical analyses

All statistical analyses were conducted with R version 4.3.0 and R Studio version 2023.03.1. Hierarchical clustering of mucus monosaccharide compositions was performed using the package *pheatmaps* and the “complete” clustering method (i.e., Euclidean distance). Permutational multivariate analysis of variance (PERMANOVA, *vegan* package, 999 permutations) was used to test for differences in Euclidean distance matrices of carbohydrate compositions between groups (i.e., clusters, clades, studies, geographic regions), and homogeneity of dispersion among groups was tested with permutational multivariate analysis of dispersion (PERMDISP, *vegan* package). Homogeneity of dispersion was not given for the factors *Clade* and *Study*, but PERMANOVA is robust to heterogeneity in dispersion for balanced designs^65^ and sample sizes for *Clade* were nearly balanced (n_2_/n_1_ = 1.33). The two studies which only reported mucus compositions for one species^27,48^ were not included in PERMANOVA analysis for the factor *Study* to reduce the heterogeneity in dispersion. Distance matrices were additionally visualized with non-metric multidimensional scaling (NMDS, *vegan* package) to display the effects of *Cluster*, *Clade*, *Study,* and *Geographic region* on carbohydrate compositions. For post-hoc analysis, multiple pairwise comparisons were conducted with the R package *pairwiseAdonis*, Bonferroni adjustment, and 999 permutations. To test for differences in the relative abundance of single monosaccharides between the three main clusters, Kruskal–Wallis-Tests were performed, and when significant (*p* < 0.05), multiple pairwise comparisons were conducted using the Dunn’s test with Bonferroni adjustment. Correlation between the two distance matrices of the hierarchical cluster dendrogram and the phylogenetic tree was tested with a Mantel test (*ade4* package), which is frequently used to compare phylogenetic trees and test for cophylogeny^66,67^. A tanglegram was created with the *dendextend* package^68^, combining the two distance matrices from mucus carbohydrate compositions and phylogenetic information. The measurement for *“Acropora* sp. (Red Sea)” was removed from the carbohydrate dendrogram after clustering with the *prune* function, as it could not be connected to a species of the phylogenetic tree.

### Supplementary Information

Supplementary Information.

## Data Availability

All data generated or analyzed during this study are included in this published article and its supplementary information files.
