# Publisher Correction: Mucus carbohydrate composition correlates with scleractinian coral phylogeny

**DOI:** 10.1038/s41598-024-67394-y

**Published:** 2024-07-17

**Authors:** Bianca M. Thobor, Arjen Tilstra, Benjamin Mueller, Andreas Haas, Jan-Hendrik Hehemann, Christian Wild

**Affiliations:** 1 Department of Marine Ecology, University of Bremen, Bremen, Germany; 2 Department of Freshwater and Marine Ecology, University of Amsterdam, Amsterdam, The Netherlands; 3 CARMABI Foundation, Willemstad, Curaçao; 4 Department of Microbiology & Biogeochemistry, NIOZ Royal Netherlands Institute for Sea Research, Texel, The Netherlands; 5 Department of Marine Glycobiology, Max Planck Institute for Marine Microbiology, Bremen, Germany; 6 MARUM Centre for Marine Environmental Sciences, University of Bremen, Bremen, Germany

Correction to: *Scientific Reports* 10.1038/s41598-024-64828-5, published online 18 June 2024

The Funding section in the original version of this Article was omitted. The Funding section now reads:

A.T. was funded by the German Research Foundation (DFG) through Grant No. Wi 2677/16-1 to C.W. J.-H.H. received funding from the Cluster of Excellence initiative (EXC-2077-390741603) and the DFG Heisenberg program (HE 7217/1-1). B.M. received funding from the European Union’s Horizon 2020 research and innovation program under the Marie Skłodowska-Curie grant (Agreement No. 894645).

The original Article has been corrected.

