# Supplementary material for: Mucus carbohydrate composition correlates with scleractinian coral phylogeny
Source: Sci Rep. 2024 Jun 18;14:14019. doi: 10.1038/s41598-024-64828-5 (PMC11189453; doi:10.1038/s41598-024-64828-5)
Supplement: Supplementary file 1 — Supplementary Information. [file 41598_2024_64828_MOESM1_ESM.pdf]

Mucus carbohydrate composition correlates with scleractinian coral phylogeny

Bianca M. Thobor[1*], Arjen Tilstra[1], Benjamin Mueller[1,2,3], Andreas Haas[4], Jan-Hendrik Hehemann[5,6], and Christian Wild[1]

[1]Department of Marine Ecology, University of Bremen, Bremen, Germany

[2]Department of Freshwater and Marine Ecology, University of Amsterdam, Amsterdam, the Netherlands

[3]CARMABI Foundation, Willemstad, Curaçao

[4]Department of Microbiology & Biogeochemistry, NIOZ Royal Netherlands Institute for Sea Research, Texel, The Netherlands

[5]Department of Marine Glycobiology, Max Planck Institute for Marine Microbiology, Bremen, Germany

[6]MARUM Centre for Marine Environmental Sciences, University of Bremen, Bremen, Germany

*Corresponding author: thobor@uni-bremen.de

Supplementary Material

Supplementary Table S1. Monosaccharide concentrations (mg L^{-1}) of hydrolyzed coral mucus from five species of scleractinian corals (raw data for Table 1). GalN = galactosamine, Xyl = xylose, Gal = galactose, Fuc = fucose, Glc = glucose, Man = mannose, Ara = arabinose, GlcN = glucosamine.

**Species**	**Rha**	**GalN**	**Xyl**	**Gal**	**Fuc**	**Glc**	**Man**	**Ara**	**GlcN**
Acropora cervicornis	0.00	1.48	0.00	2.69	0.97	0.00	14.12	24.71	16.74
Acropora cervicornis	0.00	1.51	0.00	2.57	1.02	0.00	7.03	21.27	11.16
Acropora cervicornis	0.00	1.60	0.43	2.78	1.12	0.00	9.05	22.42	13.28
Diploria labyrinthiformis	0.00	0.05	0.00	0.00	5.45	0.08	2.58	0.00	3.53
Diploria labyrinthiformis	0.00	0.00	0.52	0.00	2.22	0.00	0.56	0.00	1.59
Diploria labyrinthiformis	0.00	0.00	0.00	0.00	0.13	0.00	0.00	0.00	0.16
Meandrina meandrites	0.00	0.00	0.00	0.00	1.56	0.35	0.40	0.00	1.95
Meandrina meandrites	0.00	0.00	0.00	0.00	0.73	0.00	0.35	0.00	1.62
Montipora confusa	0.00	0.00	0.00	0.00	0.00	0.00	0.00	1.43	1.93
Montipora confusa	0.00	0.00	0.00	0.00	0.00	0.00	0.00	1.38	2.08
Montipora confusa	0.00	0.00	0.00	0.00	0.00	0.00	0.00	1.35	1.83
Montipora digitata	0.00	0.00	0.00	0.00	0.00	0.00	0.00	1.60	2.87
Montipora digitata	0.00	0.00	0.00	0.00	0.00	0.00	0.00	2.65	3.89
Montipora digitata	0.00	0.00	0.00	0.00	0.00	0.00	0.00	1.81	2.70

Supplementary Table S2. Data used in the heatmap (Fig. 1), including sources of original data and annotations concerning data preparation.

Reference	Region	Coral genus / species	Family	Clade	Replicates	Fuc	Rha	GalN /GalNAc	Ara	GlcN /GlcNAc	Gal	Glc	Man	Xyl
This study	Caribbean (Curaçao)	*Acropora cervicornis*	Acroporidae	Complex	3	2.0	0.0	2.7	48.6	24.2	4.8	0.0	17.4	0.3
This study	Caribbean (Curaçao)	*Meandrina meandrites*	Meandrinidae	Robust	2	22.4	0.0	0.0	0.0	67.7	0.0	2.7	7.2	0.0
This study	Caribbean (Curaçao)	*Diploria labyrinthiformis*	Faviidae	Robust	3	47.8	0.0	0.1	0.0	37.2	0.0	0.2	10.6	4.0
This study	From Indo-Pacific, grown in aquarium facility in Germany	*Montipora confusa*	Acroporidae	Complex	3	0.0	0.0	0.0	46.0	54.0	0.0	0.0	0.0	0.0
This study	From Indo-Pacific, grown in aquarium facility in Germany	*Montipora digitata*	Acroporidae	Complex	3	0.0	0.0	0.0	43.1	56.9	0.0	0.0	0.0	0.0
Hadaidi et al. (2019)[1]	Central Red Sea (Shaab reef)	*Acropora pharaonsis*	Acroporidae	Complex	3	1.3	1.2	0*	38.5	1.8	4.1	25.1	22.3	5.8
Hadaidi et al. (2019)[1]	Central Red Sea (Shaab reef)	*Galaxea fascicularis*	Euphyllidae	Complex	3	5.6	0.0	0*	0.0	1.6	25.1	18.3	35.1	14.3
Hadaidi et al. (2019)[1]	Central Red Sea (Shaab reef)	*Porites lobata*	Poritidae	Complex	3	11.2	0.6	0*	18.5	4.9	47.0	10.1	7.3	0.8
Hadaidi et al. (2019)[1]	Central Red Sea (Shaab reef)	*Pocillopora verrucosa*	Pocilloporidae	Robust	3	0.0	0.0	0*	0.0	0.0	4.9	33.6	45.5	19.3
Hadaidi et al. (2019)[1]	Central Red Sea (Shaab reef)	*Stylophora pistillata*	Pocilloporidae	Robust	3	0.0	0.0	0*	0.0	4.4	1.8	34.9	49.0	14.0
Lee et al. (2016)	Taiwan (Nan-wan)	*Acropora muricata*	Acroporidae	Complex	4	2.9	0*	0.7	8.0	30.2	10.5	20.9	10.4	6.1
Wild et al. (2010)	Northern Red Sea (Gulf of Aqaba)	*Acropora* sp.	Acroporidae	Complex	4-6	6.5	0.0	0.0	76.4	6.6	3.7	1.2	5.7	0.0
Wild et al. (2010)	Northern Red Sea (Gulf of Aqaba)	*Fungia* sp.[2]	Fungiidae	Robust	4-6	77.6	0.0	0.0	0.0	1.6	0.2	0.3	20.3	0.0
Wild et al. (2010)	Northern Red Sea (Gulf of Aqaba)	*Ctenactis* sp.	Fungiidae	Robust	4-6	5.2	0.0	0.0	0.0	60.8	6.0	5.9	22.1	0.0
Wild et al. (2010)	Sweden (Tisler Reef)	*Desmophyllum* sp.	Caryophylliidae	Robust	4-8	0.0	0.0	0.0	0.0	0.0	0.0	59.6	40.4	0.0
Wild et al. (2010)	Norway (Røst Reef)	*Desmophyllum* sp.	Caryophylliidae	Robust	4-8	8.0	0.0	0.0	0.0	57.2	4.7	9.8	18.8	1.5
Wild et al. (2010)	Norway (Røst Reef)	*Madrepora* sp.	Oculinidae	Robust	4-8	0.0	31.4	0.0	0.0	0.0	0.0	26.0	42.6	0.0
Wild et al. (2010)	Northern Red Sea (Gulf of Aqaba)	*Pocillopora* sp.	Pocilloporidae	Robust	4-6	25.3	0.0	0.0	0.0	0.0	0.0	25.2	49.5	0.0
Wild et al. (2010)	Northern Red Sea (Gulf of Aqaba)	*Stylophora* sp.[3]	Pocilloporidae	Robust	4-6	0.0	0.0	0.0	0.0	0.0	0.0	100.0	0.0	0.0
Klaus et al. (2007)[1]	Caribbean (Curaçao)	*Orbicella annularis*	Merulinidae	Robust	36	38.5	0.0	0*	0.2	46.9	4.7	8.8	0.0	0.8
Wild et al. (2005)	Great Barrier Reef (Heron Island)	*Acropora pulchra*	Acroporidae	Complex	3	7.8	0.0	0*	25.4	10.7	2.9	32.2	11.1	9.8
Wild et al. (2005)	Great Barrier Reef (Heron Island)	*Acropora digitifera*	Acroporidae	Complex	3	5.0	2.8	0*	13.9	16.4	5.3	40.5	12.0	4.0
Wild et al. (2005)	Great Barrier Reef (Heron Island)	*Acropora robusta*	Acroporidae	Complex	3	6.6	8.0	0*	24.9	10.6	5.9	22.1	13.4	4.7
Wild et al. (2005)	Great Barrier Reef (Heron Island)	*Acropora aspera*	Acroporidae	Complex	3	5.5	0.0	0*	50.8	13.7	6.2	13.2	10.6	0.0
Wild et al. (2005)	Great Barrier Reef (Heron Island)	*Acropora millepora*	Acroporidae	Complex	3	0.0	0.0	0*	63.2	7.9	5.3	12.5	11.1	0.0
Wild et al. (2005)	Great Barrier Reef (Heron Island)	cf. *Acropora muricata*	Acroporidae	Complex	3	5.6	0.0	0*	36.7	17.2	5.4	22.2	12.8	0.0
Meikle et al. (1988)	Great Barrier Reef (Magnetic Island)	*Acropora muricata*	Acroporidae	Complex	1	2.0	0*	1.0	47.0	29.0	2.0	1.0	18.0	0.0
Meikle et al. (1988)	Great Barrier Reef (Magnetic Island)	*Pachyseris speciosa*	Agariciidae	Complex	1	14.0	0*	2.0	16.0	10.0	46.0	0.0	12.0	0.0
Meikle et al. (1988)	Great Barrier Reef (Magnetic Island)	*Fungia fungites*	Fungiidae	Robust	1	41.0	0*	7.0	2.0	22.0	4.0	3.0	19.0	2.0

*Study did not report the absence of GalN/GalNAc or Rha, but value was set to zero because the method used (see Table S1) generally enables the detection.

[1] Study included additional monosaccharides which were not included in the analysis of the present study, and mole % values were adjusted accordingly.

[2] Three separate measurements of *Fungia* sp. from different seasons were averaged.

[3] *Stylophora* sp. was not included in the heatmap (Fig. 1) because only Glc was detected, indicating low carbohydrate concentrations in general, which likely lead to over-estimation of the relative proportion of Glc.

Supplementary Table S3. Overview of methods for mucus collection and monosaccharide measurement. PAD = pulsed amperometric detector; HPLC = high performance liquid chromatography; HPAEC = high-performance anion exchange chromatography; GC = gas chromatography; GC-MS = combined gas chromatography/ mass spectrometry; TMS = per-O-trimethylsilyl.

Reference	Mucus collection	Measurement of monosaccharides
Meikle et al. (1988)	Drawn from the colony surface after chilling corals for 20 h at 4 °C under toluene	Dialysis, centrifugation, acid hydrolysis of the supernatant, GC-MS of alditol acetate derivatives
Wild et al (2005)	"milked" colonies by exposing them to air for 2 minutes and collecting the dripping mucus	Dialysis (10 kDa), lyophilization, acid hydrolysis, GC-MS of TMS derivatives, analysis done at the Center of Complex Carbohydrate Research (CCCR) of the University of Georgia in Athens, Georgia, USA
Klaus et al. (2007)	Drawn from the colony surface	Dialysis (1 kDa), acid hydrolysis, HPAEC-PAD
Wild et al (2010)	"milked" for 2 minutes	Dialysis (100-500 Da), lyophilization, acid methanolysis, GC-MS of TMS derivatives, analysis done at the CCCR
Lee at al. (2016)	"milked" for 5 minutes	Dialysis (100-500 Da), acid hydrolysis, HPLC-MS of TMS derivatives
Hadaidi et al. (2019)	Drawn from colony surface *in situ*	Dialysis (50 kDa), acid methanolysis, GC-MS of TMS derivatives, analysis done at the CCCR
Present study	"milked" for 2 minutes	Acid hydrolysis, 1:100 dilution, centrifugation, HPAEC-PAD

Supplementary Table S4. Species list for phylogenetic tree based on mitochondrial COI (cytochrome *c* oxidase subunit I) gene.

Species	Location	Length (bp)	Accession no.*	Reference
Acropora aspera	Unknown	657 bp	KX664114	Unpublished
Acropora cervicornis	Caribbean	658 bp	AY451340	(Shearer and Coffroth, 2008)
Acropora digitifera	Unknown	681 bp	KR401100	Unpublished
Acropora millepora	Sabah, Malaysia	658 bp	MG383848	(Robert et al., 2019)
Acropora muricata	Sabah, Malaysia	657 bp	KX664143	Unpublished
Acropora pharaonis	Unknown	664 bp	MK309942	Unpublished
Acropora pulchra	Sabah, Malaysia	658 bp	MG383851	(Robert et al., 2019)
Acropora robusta	Unknown	590 bp	MN413872	Unpublished
Ctenactis crassa	Indo-Pacific	504 bp	LC191439	(Oku et al., 2017)
Desmophyllum dianthus	Mediterranean	512 bp	JQ611389	(Addamo et al., 2012)
Diploria labyrinthiformis	Atlantic	630 bp	AB117224	(Fukami et al., 2004)
Fungia fungites	Nansei Island group, southern Japan	505 bp	LC484536	(Oku et al., 2020)
Galaxea fascicularis	Unknown	590 bp	MN413859	Unpublished
Madrepora oculata	Mediterranean	512 bp	JQ611395	(Addamo et al., 2012)
Meandrina meandrites	Atlantic	630 bp	AB117296	(Fukami et al., 2004)
Montipora undata	Sodwana Bay, South-Africa	590 bp	MN413817	Unpublished
Montipora digitata	Indo-Pacific	657 bp	KF492662	(Swain et al., 2016)
Orbicella annularis	Caribbean	658 bp	AY451352	(Shearer and Coffroth, 2008)
Pachyseris speciosa	Unknown	590 bp	MN413852	Unpublished
Pocillopora verrucosa	Unknown	590 bp	MN413871	Unpublished
Porites lobata	Islas Marias Biosphere Reserve, Mexico	627 bp	MN005655	(Santiago-Valentín et al., 2020)
Stylophora pistillata	Unknown	590 bp	MN413851	Unpublished

bp = basepairs
*All sequences were obtained from the NCBI GenBank database

References

Addamo, A.M., Reimer, J.D., Taviani, M., Freiwald, A., Machordom, A., 2012. *Desmophyllum dianthus* (Esper, 1794) in the scleractinian phylogeny and its intraspecific diversity. PLoS ONE 7, e50215. https://doi.org/10.1371/journal.pone.0050215

Fukami, H., Budd, A.F., Paulay, G., Solé-Cava, A., Allen Chen, C., Iwao, K., Knowlton, N., 2004. Conventional taxonomy obscures deep divergence between Pacific and Atlantic corals. Nature 427, 832–835. https://doi.org/10.1038/nature02339

Hadaidi, G., Gegner, H.M., Ziegler, M., Voolstra, C.R., 2019. Carbohydrate composition of mucus from scleractinian corals from the central Red Sea. Coral Reefs 38, 21–27. https://doi.org/10.1007/s00338-018-01758-5

Klaus, J.S., Janse, I., Heikoop, J.M., Sanford, R.A., Fouke, B.W., 2007. Coral microbial communities, zooxanthellae and mucus along gradients of seawater depth and coastal pollution. Environ. Microbiol. 9, 1291–1305. https://doi.org/10.1111/j.1462-2920.2007.01249.x

Lee, S.T.M., Davy, S.K., Tang, S.-L., Kench, P.S., 2016. Mucus sugar content shapes the bacterial community structure in thermally stressed *Acropora muricata*. Front. Microbiol. 7, 371. https://doi.org/10.3389/fmicb.2016.00371

Meikle, P., Richards, G.N., Yellowlees, D., 1988. Structural investigations on the mucus from six species of coral. Mar. Biol. 99, 187–193. https://doi.org/10.1007/BF00391980

Oku, Y., Iwao, K., Hoeksema, B.W., Dewa, N., Tachikawa, H., Koido, T., Fukami, H., 2020. *Fungia fungites* (Linnaeus, 1758) (Scleractinia, Fungiidae) is a species complex that conceals large phenotypic variation and a previously unrecognized genus. Contrib. Zool. 89, 188–209. https://doi.org/10.1163/18759866-20191421

Oku, Y., Naruse, T., Fukami, H., 2017. Morpho-molecular evidence for polymorphism in the mushroom coral *Cycloseris hexagonalis* (Scleractinia: Fungiidae), with a new phylogenetic position and the establishment of a new genus for the species. Zoolog. Sci. 34, 242–251. https://doi.org/10.2108/zs160065

Robert, R., Rodrigues, K.F., Waheed, Z., Kumar, S.V., 2019. Extensive sharing of mitochondrial *COI* and *CYB* haplotypes among reef-building staghorn corals (*Acropora* spp.) in Sabah, North Borneo. Mitochondrial DNA Part A 30, 16–23. https://doi.org/10.1080/24701394.2018.1448080

Santiago-Valentín, J.D., Rodríguez-Troncoso, A.P., Bautista-Guerrero, E., López-Pérez, A., Cupul-Magaña, A.L., 2020. Settlement ecology of scleractinian corals of the northeastern tropical Pacific. Coral Reefs 39, 133–146. https://doi.org/10.1007/s00338-019-01872-y

Shearer, T.L., Coffroth, M.A., 2008. DNA barcoding: barcoding corals: limited by interspecific divergence, not intraspecific variation: DNA barcoding. Mol. Ecol. Resour. 8, 247–255. https://doi.org/10.1111/j.1471-8286.2007.01996.x

Swain, T.D., DuBois, E., Gomes, A., Stoyneva, V.P., Radosevich, A.J., Henss, J., Wagner, M.E., Derbas, J., Grooms, H.W., Velazquez, E.M., Traub, J., Kennedy, B.J., Grigorescu, A.A., Westneat, M.W., Sanborn, K., Levine, S., Schick, M., Parsons, G., Biggs, B.C., Rogers, J.D., Backman, V., Marcelino, L.A., 2016. Skeletal light-scattering accelerates bleaching response in reef-building corals. BMC Ecol. 16, 10. https://doi.org/10.1186/s12898-016-0061-4

Wild, C., Naumann, M., Niggl, W., Haas, A., 2010. Carbohydrate composition of mucus released by scleractinian warm- and cold-water reef corals. Aquat. Biol. 10, 41–45. https://doi.org/10.3354/ab00269

Wild, C., Woyt, H., Huettel, M., 2005. Influence of coral mucus on nutrient fluxes in carbonate sands. Mar. Ecol. Prog. Ser. 287, 87–98. https://doi.org/10.3354/meps287087
